# Broad Fungal Compatibility and Seed Size May Facilitate Invasiveness in Two Asian Terrestrial Orchids *Spathoglottis plicata* and *Arundina graminifolia*

**DOI:** 10.1002/ece3.73805

**Published:** 2026-06-10

**Authors:** Tomáš Figura, Edita Tylová, František Novák, Vincent S. F. T. Merckx, Jan Ponert, Julita Minasiewicz, Marc‐André Selosse, Florent Martos

**Affiliations:** ^1^ Institute of Botany Czech Academy of Sciences Průhonice Czech Republic; ^2^ Department of Experimental Plant Biology, Faculty of Science Charles University Prague Czech Republic; ^3^ Naturalis Biodiversity Center Leiden the Netherlands; ^4^ Department of Evolutionary and Population Biology, Institute for Biodiversity and Ecosystem Dynamics University of Amsterdam Amsterdam the Netherlands; ^5^ Department of Plant Taxonomy and Nature Conservation, Faculty of Biology University of Gdańsk Gdańsk Poland; ^6^ Institut Systématique Evolution Biodiversité (ISYEB), Muséum National d'Histoire Naturelle, CNRS Sorbonne Université, EPHE, Université des Antilles Paris France

**Keywords:** biological invasions, orchid mycorrhizal fungi, Orchidaceae, symbiotic seed germination, Tulasnellaceae

## Abstract

Two Asian orchids, the bamboo orchid ( *Arundina graminifolia* ) and the Philippine ground orchid ( *Spathoglottis plicata* ), are expanding into South and Central America, the Pacific, and Africa. Here, we tested whether seed traits and low in vitro selectivity toward mycorrhizal fungi may contribute to their successful spread outside the native range. We examined seed size and reserves, asymbiotic and symbiotic germination with orchid mycorrhizal fungi from different geographic regions. Both species have relatively large seeds, with *A. graminifolia* having some of the largest among orchids. Seeds contain lipids, proteins, and low amounts of soluble saccharides and starch. Despite these reserves, both orchids are initially mycoheterotrophic and require an external carbon source for early development. In asymbiotic culture, both species showed high germination on sucrose‐containing media, with maximum germination exceeding 95%. In symbiotic culture, both species formed protocorms with Tulasnellaceae isolates from different continents, including isolates obtained from locations separated by more than 9000 km. This capacity was broader in *S. plicata* . Our results suggest that the success of *S. plicata* outside its native range may be facilitated by broad compatibility with Tulasnellaceae, some of which have a global distribution, and partly by self‐pollination. In *A. graminifolia* , relatively large seeds may provide greater internal reserves for early development, but this species appears more specific toward certain Tulasnellaceae lineages and remains pollinator‐dependent. Together with the ability to colonize disturbed habitats, these traits may help explain the successful spread of both orchids outside their native range.

## Graphical abstract

Two Asian orchids, *Arundina graminifolia* and *Spathoglottis plicata* , are expanding into tropical regions worldwide, showing invasive behavior. In this study, we investigated the factors underlying their success. Both species—as all orchids—require fungal carbon to germinate, but *S. plicata* associates with a broader range of Tulasnellaceae fungi from different continents, which likely facilitates its global spread. In contrast, *A. graminifolia* produces larger seeds with greater reserves but forms more specific fungal partnerships, suggesting different strategies underlying their invasiveness.

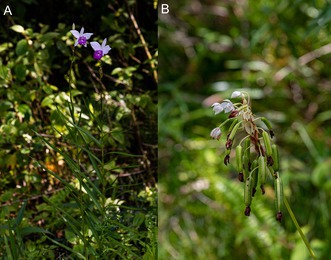

## 1 Introduction

Alien species may perish, become naturalized, or flourish as successful invaders in novel habitats (Richardson et al. 2000). Biotic invasions represent one of the most severe threats to global biodiversity (Mack et al. 2000), particularly on oceanic islands (Kueffer et al. 2010). Plant invasions, driven by climate change and human activities, including globalization, are increasing globally and causing significant ecological and economic harm (Gioria et al. 2024). Invasive plants are typically generalists in terms of ecological interactions, as specific interactions are rarely replicated when a species is introduced outside its native range (Richardson et al. 2000).

Although Darwin (1859) noted the extreme colonization potential of orchids due to enormous production of small seeds, these plants are usually considered rare and ecologically sensitive. Orchid seeds cannot germinate autonomously and often cease growth at the protocorm stage without an external carbon source. They rely on an external carbon source for successful germination and further growth. Therefore, all orchids depend on carbon provided by fungi, at least during early ontogeny (Rasmussen 1995). In asymbiotic germination experiments in vitro, carbon is mostly supplied as soluble carbohydrates in the culture media, often allowing very high, nearly 100% germination rates (Figura et al. 2019; Kauth et al. 2008; Ponert et al. 2011). This dependence on external fungal carbon during germination is called initial mycoheterotrophy and orchids may later become autotrophic, remain obligately dependent on fungi (mycoheterotrophic), or stay somewhere in between these two extremes—mixotrophic (Merckx et al. 2024). This dependency, in fact, should limit their ability to colonize new environments, as their survival and growth are closely tied to the availability and compatibility of fungal partners.

Unlike the majority of plants, initial mycoheterotrophs, including all orchids, have very small seeds called dust seeds, with very limited reserves (Eriksson and Kainulainen 2011; Figura et al. 2019, 2024; Selosse et al. 2025). Often, these small seeds consist of a dead testa filled with air, while tiny embryos usually occupy only a small part of the seed (Arditti and Ghani 2000; Yeung 2017). Orchid embryos have no obvious histodifferentiation, except for the suspensor, which is sometimes present and may even serve as a source of nutrition (Yeung 2017). Root and shoot meristems, cotyledons, and even endosperm are missing in orchid seeds (Lee and Yeung 2018). This is an even greater reduction than in some other dust‐seeded plants (Figura et al. 2019). Rarely, a gradient of cell size is observed within the embryo, with smaller cells at the chalazal pole and larger cells at the micropylar pole, hosting the fungi (Lee and Yeung 2018; Rasmussen 1990). However, in approximately 10 orchid species, rudiments of cotyledons have been reported (Nishimura 1991; Yeung 2017). Some authors consider embryos with a spindle shape as cotyledonous, and this group includes *Arundina graminifolia* among others (Nishimura 1991; Vinogradova and Andronova 2002). Embryo size varies widely among Orchidaceae, from approximately 8 cells in *Epipogium aphyllum* to 700 in *Bletilla striata* , which is considered the biggest orchid embryo (Rasmussen 1995; Veyret 1974). Although all orchids produce extremely small seeds, their sizes and the amount of reserves vary significantly among species (Arditti and Ghani 2000; Lee and Yeung 2018). Given the minute size of orchid seeds, their highly reduced embryos, and limited reserves, assessing embryo development and reserve composition may help clarify whether these traits are associated with early germination and establishment‐related responses.

We may even speculate that more developed embryos and seeds with greater storage reserves may facilitate germination and, in the case of orchids, enhance their potential for invasiveness.

To germinate, the vast majority of orchids form associations with a polyphyletic group of fungi comprising Ceratobasidiaceae, Tulasnellaceae and Serendipitaceae, collectively referred to as “rhizoctonias,” while others establish connections with ectomycorrhizal fungi and non‐rhizoctonia saprotrophs (Figura et al. 2021; Selosse et al. 2022). Stable isotope analyses indicate that rhizoctonia‐associated orchids are less dependent on fungal carbon at the adult stage than ectomycorrhizal‐associated orchids (Cameron et al. 2008; Ogura‐Tsujita and Yukawa 2008; Suetsugu, Matsubayashi, and Okada 2025; Suetsugu, Yagi, et al. 2025).

There is considerable variation in the level of specificity of orchid species toward mycorrhizal fungi, ranging from a broad generalism where a species can associate with various phylogenetically distant fungi to strict specificity toward a single fungal strain (Bidartondo et al. 2004; Girlanda et al. 2006; Jacquemyn et al. 2021; Waud et al. 2017). Strict specificity is often observed in highly fungus‐dependent plants (Girlanda et al. 2006; Matsuda et al. 2020; Ogura‐Tsujita et al. 2012; Suetsugu et al. 2022; Zimmer et al. 2007).

Despite the prevailing view that orchids occupy narrow ecological niches and exhibit slow growth rates, there are exceptions. Out of approximately 28,000 orchid species, around 90 are considered weedy or even invasive (Kolanowska and Konowalik 2014), representing approximately 0.3% of the diversity of this family.

Two such species, the bamboo orchid ( *A. graminifolia* ) and the Philippine orchid ( *Spathoglottis plicata* ), are native to southeast Asia. Due to their ornamental value, they were introduced outside their native range, where they have spread rapidly, particularly *S. plicata* . The most affected regions include South and Central America, parts of Africa, the Mascarene and Caribbean Islands, and Hawaii. The spread of these orchids is likely facilitated by their ability to colonize ruderal and disturbed habitats such as roadsides, road cuts, abandoned fields, lava fields, etc. and rapid spread to new areas (Ackerman et al. 2014; Clifford and Kobayashi 2012; Kolanowska and Konowalik 2014). For example, in Puerto Rico, *S. plicata* occupies the same habitats as the native orchid *Bletia patula* , decreasing its reproductive success indirectly by increasing populations of florivorous weevil (Recart et al. 2013). Furthermore, this species has formed a symbiotic relationship with ants, which are attracted to its nectaries and prey on weevils (Ackerman et al. 2014).

In contrast to rarer species, common or fast spreading weedy orchids usually form relationships with a wide array of fungi (Bonnardeaux et al. 2007; Ogura‐Tsujita and Yukawa 2008) and associate with rhizoctonias (Taylor and Bruns 1997), although there are exceptions (Ogura‐Tsujita and Yukawa 2008).

Ackerman et al. (2014) suggested that *S. plicata* is likely a generalist with respect to mycorrhizal fungi. Indeed, morphological determination of the fungi isolated from its roots showed associations with several different rhizoctonia groups (Athipunyakom et al. 2004). Other studies found only Tulasnellaceae (Aewsakul et al. 2013; Meng et al. 2019), including in the roots of a close relative, *Spathoglottis pubescens* (Shan et al. 2002).

Molecular and morphological determination showed that *A. graminifolia* can also germinate with *Tulasnella* (Meng et al. 2019), consistent with an earlier study by Ma et al. (2003) and Shan et al. (2002). In addition, *A. graminifolia* has been found to be specific toward Tulasnellaceae outside its native range in Brazil (Pereira Angeli 2019). However, it remains unknown whether invasive orchids can utilize the pool of symbiotic fungi available in newly colonized areas.

Weedy orchids are also capable of thriving in diverse habitats, and it can be expected that they have broad pollinator associations. For example, *A. graminifolia* and *S. plicata* are pollinated by a wide range of globally distributed pollinators. Additionally, unlike *A. graminifolia* , *S. plicata* is even capable of self‐pollination, increasing its pollination success (Ackerman et al. 2014; Sugiura 2014). These pollination traits are relevant to invasion because reliable seed production increases propagule pressure and thus the likelihood of establishment. Combined with high germination capacity and broad mycorrhizal compatibility, they may further promote colonization in novel environments.

Thus, in this study, we investigate the reserves contained in the seeds of *A. graminifolia* and *S. plicata* , and their in vitro capacity to germinate asymbiotically or symbiotically with different species and clades of rhizoctonia fungi. We address the following questions: (i) Do their mature seeds contain unusually high amounts of storage compounds, enhancing their ability to germinate without fungus? (ii) Do their seeds depend on a mycorrhizal fungus to germinate and grow into plantlets? (iii) Which carbon sources can be utilized by these orchids for germination? (iv) Do they exhibit similar germination ability with different mycorrhizal fungi from diverse geographical origins? When seeds from different continents are available, do they germinate as well as local seeds with local fungi? By answering these questions, we aim to identify the factors driving the expansion of these species beyond their native range.

## 2 Materials and Methods

### 2.1 Plant Material

**FIGURE 1:**
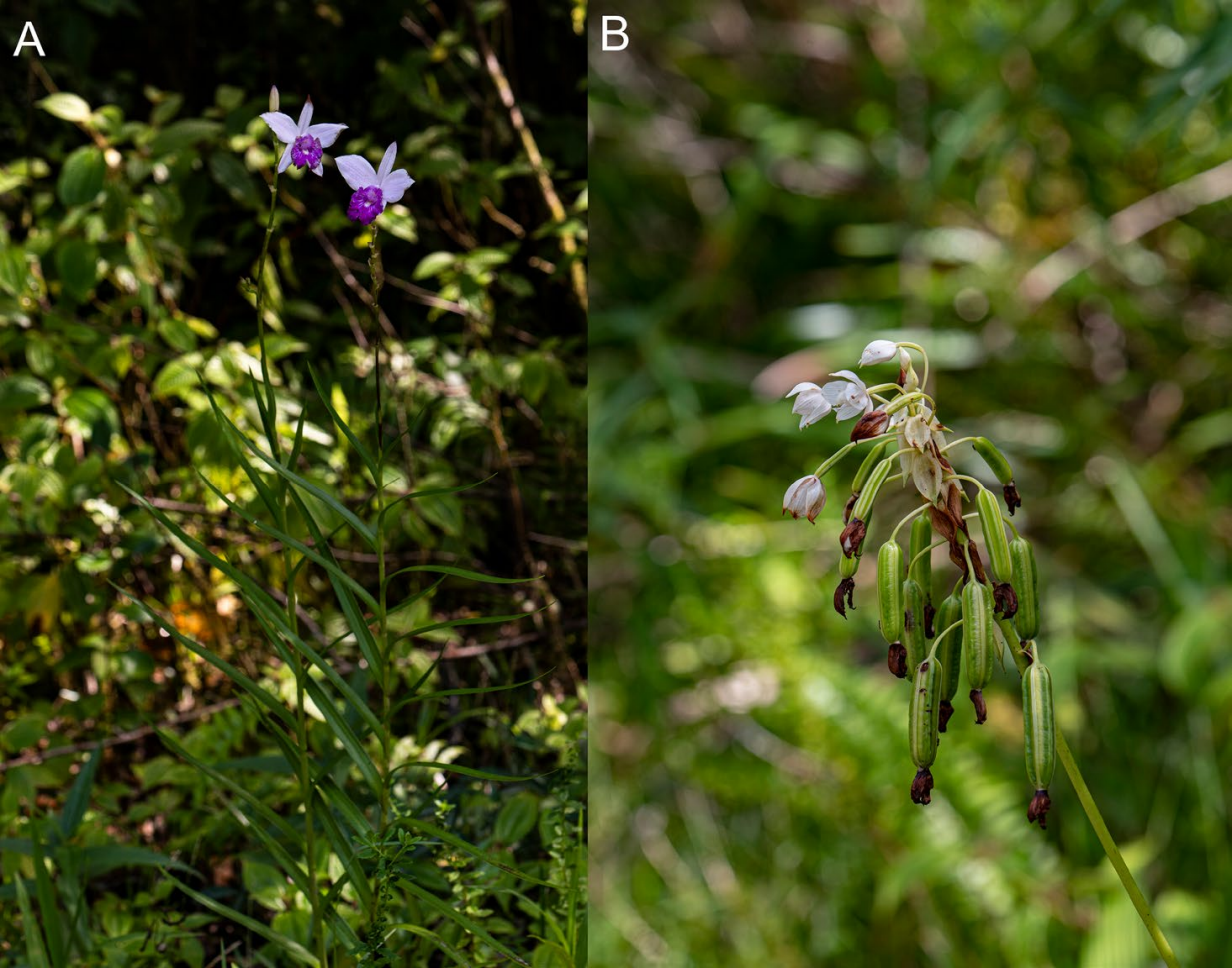
Studied plant species (A) *Arundina graminifolia* and (B) *Spathoglottis plicata* in typical ruderal habitats around Saint‐Philippe, La Réunion.

Ripe seeds of *A. graminifolia* and *S. plicata* (Figure 1) were collected on the oceanic island La Réunion, St. Philippe, France, 21°21′44.138″ S, 55°45′52.326″ E, 5 m a.s.l., on 15 March 2023 into paper bags. Seeds of *A. graminifolia* were also collected at Dapa, Colombia, 3°33′19.545″ N, 76°33′9.943″ W, at 1620 m a.s.l., on 22 August 2023, also fully ripened. The second collection site was selected in order to test symbiotic germination of seeds from different geographical origins. The main locality, St. Philippe in La Réunion, is a lowland tropical rainforest area below 100 m a.s.l. It is characterized by ruderal vegetation, an average annual temperature of 25°C, and average annual rainfall of up to 10,000 mm per year. In this area, both species are also found on the edges of preserved natural or secondary forest, whilst *Spathoglottis* also colonizes the herbaceous vegetation of early successional stages on young lava flows. The Colombian locality is a garden with a drier climate, at approx. 1600 m a.s.l.

### 2.2 Fungal Material

Fungi were isolated following the protocol described in Mennicken et al. (2024). Some isolates were newly obtained in this study, whereas others were taken from previous studies. Isolations of new fungi for this study were performed from orchid roots collected in La Réunion and Poland to test the performance of mycorrhizal fungi from different continents. In addition, fungal isolates obtained from orchid roots in Europe in previous studies were included (Mennicken et al. 2024; Figura et al. 2021, Table S2).

Because we finally used several isolates of Tulasnellaceae and the classification of this group is quite complex (e.g., Arifin et al. 2022), we performed phylogenetic analysis of Tulasnellaceae to understand relationships of our isolates with available sequences. All ITS sequences newly obtained in this study from Tulasnellaceae (for details see Table S2) were compared to the GenBank database and all closely related matches were used to build the alignment. We also included sequences of named *Tulasnella* species which were used in previous phylogenetic analyses of orchid associates (Arifin et al. 2022; Cruz et al. 2014; Freitas et al. 2020; Linde et al. 2017; Nguyen et al. 2020; Reiter et al. 2023) and representatives of all five lineages identified by Cruz et al. (2014) and Arifin et al. (2022). In total, we obtained 103 ITS sequences, which were used to construct a phylogeny and place our isolates within the main lineages of Tulasnellaceae defined in (Arifin et al. 2022; Cruz et al. 2014). Sequences were aligned in MAFFT using the Q‐INS‐i method (Katoh and Toh 2008) and the alignment was further checked and corrected manually. The phylogenetic tree was calculated in MEGA12 software (Kumar et al. 2024) using the Maximum Likelihood method and Kimura 2‐parameter model (Kimura 1980) of nucleotide substitutions (selected based on Bayesian Information Criterion and Akaike Information Criterion) and the tree with the highest log likelihood (−8250.60) was selected. The evolutionary rate differences among sites were modeled using a discrete Gamma distribution across 5 categories (+G, parameter = 0.7666) and, due to a well‐known extreme heterogeneity of ITS sequences in *Tulasnella* (e.g., Arifin et al. 2022), we used all alignment positions with at least 80% site coverage (comprising 440 sites). Bootstrap values are based on 1000 replicates.

### 2.3 Anatomical Analyses of Mature Seeds

To examine the presence of storage compounds in embryos, we used histochemical detection on sections and brightfield microscopy. Mature seeds of *A. graminifolia* and *S. plicata* were sectioned using a Shandon cryomicrotome after cryoprotection by incubation in 2% sucrose for 12 h at 4°C. Sections (20 μm) were collected on aluminum gelatin‐coated slides (Soukup and Tylová 2014) and stained with Sudan Red 7B (Soukup 2019) for lipid detection or Lugol's solution (1 min) for starch detection. Proteins were detected using Ponceau 2R in 2% acetic acid (10 min) and Azur (10 s) according to Gutmann et al. (1996) on paraffin sections (10 μm). Paraffin embedding followed the procedure described in Soukup and Tylová (2019), and seed sections were prepared using Leica RM 2155 microtome. Sections were documented with an Olympus BX51 microscope (Olympus Corp., Tokyo, Japan) equipped with a Nikon Digital Sight 10 camera (Nikon Europe B.V.). Embryo and testa dimensions were measured on the whole‐mount preparations of mature seeds in NaI‐based clearing solution of high refractive index (Soukup and Tylová 2019) using NIS Elements AR 3.22.15 (Laboratory Imaging, Prague, Czech Republic). Seed shape was documented by scanning electron microscopy (SEM). For SEM, mature seeds were coated with gold (2 nm thin layer) in an ion sputter device (Bal‐Tec SCD 050) and observed using a JEOL JSM‐IT 200 microscope. Seed length and width were measured using FIJI 1.54. Finally, seed weight of both species was measured using a Mettler Toledo MX5 microbalance.

### 2.4 Quantification of Soluble Saccharides and Starch in Seeds by High‐Performance Liquid Chromatography (HPLC)

Soluble saccharides and starch were extracted, identified, and quantified following the procedure of Eliášová et al. (2022). Seeds (10–15 mg) were freeze‐dried, then boiled with 80% (v/v) methanol (0.5 mL) at 75°C for 15 min. The solvent was vacuum‐evaporated and the residue was resuspended in Milli‐Q ultrapure water (Millipore). Soluble saccharides were analyzed using HPLC with refractometric detection and SP0810 Pb^2+^ column (Shodex, Tokyo, Japan), mobile phase: ultrapure water. Pellets remaining after the extraction of soluble carbohydrates were washed with ultrapure water, then starch was hydrolyzed by α‐amylase (Fluka Sigma‐Aldrich, St. Louis, USA) and amyloglucosidase (Fluka Sigma‐Aldrich, St. Louis, USA) as described by Ponert et al. (2021). The released glucose was quantified by HPLC. The amount of starch was expressed as the glucose amount after enzymatic cleavage. Only two samples were prepared per species as thousands of seeds are necessary for a single sample.

### 2.5 Seed Germination Experiments

Seeds of both species were disinfected in 5 mL syringes as previously described (Figura et al. 2019; Ponert et al. 2013). Briefly, seeds were preincubated in 70% ethanol for 5 min, washed three times with deionized water (< 0.2 μS.cm^−1^), and treated in Ca(OCl)_2_ solution for 5 min. Finally, seeds were washed three times with sterile deionized water. The Ca(OCl)_2_ solution was prepared by dissolving 20 g of chlorinated lime (Kittfort, Czech Republic) in 100 mL of deionized water, filtering through filter paper and adding a drop of Tween 20. This solution was used within 30 min after filtering to prevent coagulation. Five Petri dishes sealed with air‐permeable foil (Parafilm M) were prepared for each experimental treatment.

### 2.6 Asymbiotic In Vitro Cultivation on Different Carbon Sources

To determine whether the tested orchid species require external carbon sources for asymbiotic germination, we conducted a controlled in vitro experiment using modified culture media. The aim was to assess their ability to germinate in the absence of fungal‐derived carbon and to identify which compounds, if any, are necessary to support this process. We used BM‐1 and ¼‐2 media because preliminary experiments showed that both were suitable for asymbiotic germination of both orchids and pyroloids (Figura et al. 2019; Van Waes et al. 1986; see Table S1). These media were therefore used as positive asymbiotic controls. To evaluate the effect of different carbon sources, we prepared a modified BM‐1 medium based on Figura et al. (2024). The BM‐1 medium contains several potential carbon sources, namely sucrose, glutamine, glycine, and casein, and this combination is suitable for the tested orchids. Thus, we have prepared five variants of this medium with different carbon sources: no carbon source (“BM−”), glutamine, glycine, and casein (“BMaa”), excess glutamine, glycine, and casein (“BMaa+”), sucrose (“BMs”), and a variant containing all components (“BMfull”) (for details see Table S1). Since the “BMaa+” variant was lethal to seeds of both species, as in previous studies (Figura et al. 2024), this treatment was excluded from further experiments.

### 2.7 Symbiotic Germination of Seeds With Different Fungi From Different Regions

To test the symbiotic germination of both orchid species with fungal isolates from different geographic origins, we used a symbiotic OMA medium (10 g of agar and 3 g of oatmeal per liter, as described by Figura et al. 2021), inoculated with fungi. A non‐inoculated OMA medium served as a negative control. For the detailed composition of the media, see Table S1. After adjusting the pH to 5.8 using 1 M KOH and 0.2 M HCl, the media were autoclaved at 144 kPa, 121°C (Tuttnauer 2540 EK‐N) for 20 min and poured into 9 cm plastic Petri dishes.

For the symbiotic cultures, a 0.5 cm^2^ agar piece containing the desired fungal isolate was placed in each Petri dish. We tested the effects of 18 fungal isolates in pure cultures, including 5 Ceratobasidiaceae, 2 Serendipitaceae isolated from Europe, and 11 Tulasnellaceae (6 from La Réunion and 5 from Europe), isolated within this study or in previous studies from terrestrial orchids (Mennicken et al. 2024; Figura et al. 2020). For a complete list of tested fungal isolates, see Table S2.

To also test the symbiotic germination of seeds from different geographical origins, *A. graminifolia* seeds from both Colombia and La Réunion were sown with 6 Tulasnellaceae fungal isolates from La Réunion.

### 2.8 Cultivation Conditions

All asymbiotic and symbiotic cultures were incubated in the dark at 28°C for 3 months.

There were two exceptions: (1) The experiment with seeds from different geographical regions was evaluated earlier, after 60 days of incubation, because contamination was severe and we no longer had sufficient South American seeds to repeat the full 3‐month experiment. At that time, a sufficient number of replicates was still available. (2) In the asymbiotic cultivation experiment, the “BMaa+” medium variant was excluded from further analysis, as it proved lethal to protocorms.

### 2.9 Monitoring of Germination and Data Analysis

Cultures were observed monthly using a Zeiss Stemi 305 stereomicroscope. To assess protocorm formation rate and germination, seeds without embryos or clearly underdeveloped seeds were excluded. Only well‐developed seeds with a ruptured testa and forming rhizoids were considered protocorms (e.g., Figures S1A,O,P,R,S,U and S2M–R), following Figura et al. (2019). Each Petri dish was treated as the experimental unit. Additionally, we measured the germination percentage, which includes all seeds with a ruptured testa, including those without rhizoid formation (e.g., Figures S1K,Q and S2D,S). Images of seedlings were taken using a Canon EOS 60D equipped with a Canon EF 100 mm f/2.8 L macro lens. Germination was recorded both 60 and 90 days after sowing. Statistical analyses were performed in R (R Core Team 2021) using RStudio's 2023.09.1 + 494 “Desert Sunflower” release. When these assumptions were met, germination data were analyzed using ANOVA; otherwise, a Kruskal–Wallis test was applied (Kruskal and Wallis 1952), followed by pairwise Wilcoxon rank‐sum tests (Wilcoxon 1945). Maximum seedling (protocorm) diameter was measured using FIJI 1.54j software using the straight line, segmented line, and measure functions. Normality of the data was assessed using the Shapiro–Wilk test (Shapiro and Wilk 1965), and homogeneity of variances was assessed using the Bartlett test (Bartlett 1937). ANOVA with a nested factor (Petri dish) was used to analyze the maximum protocorm diameter. For pairwise comparisons between treatments, the ANOVA was followed by the Tukey–Kramer test (Kramer 1956).

## 3 Results

### 3.1 Morphometric Characteristics of Seeds and Embryos

**FIGURE 2:**
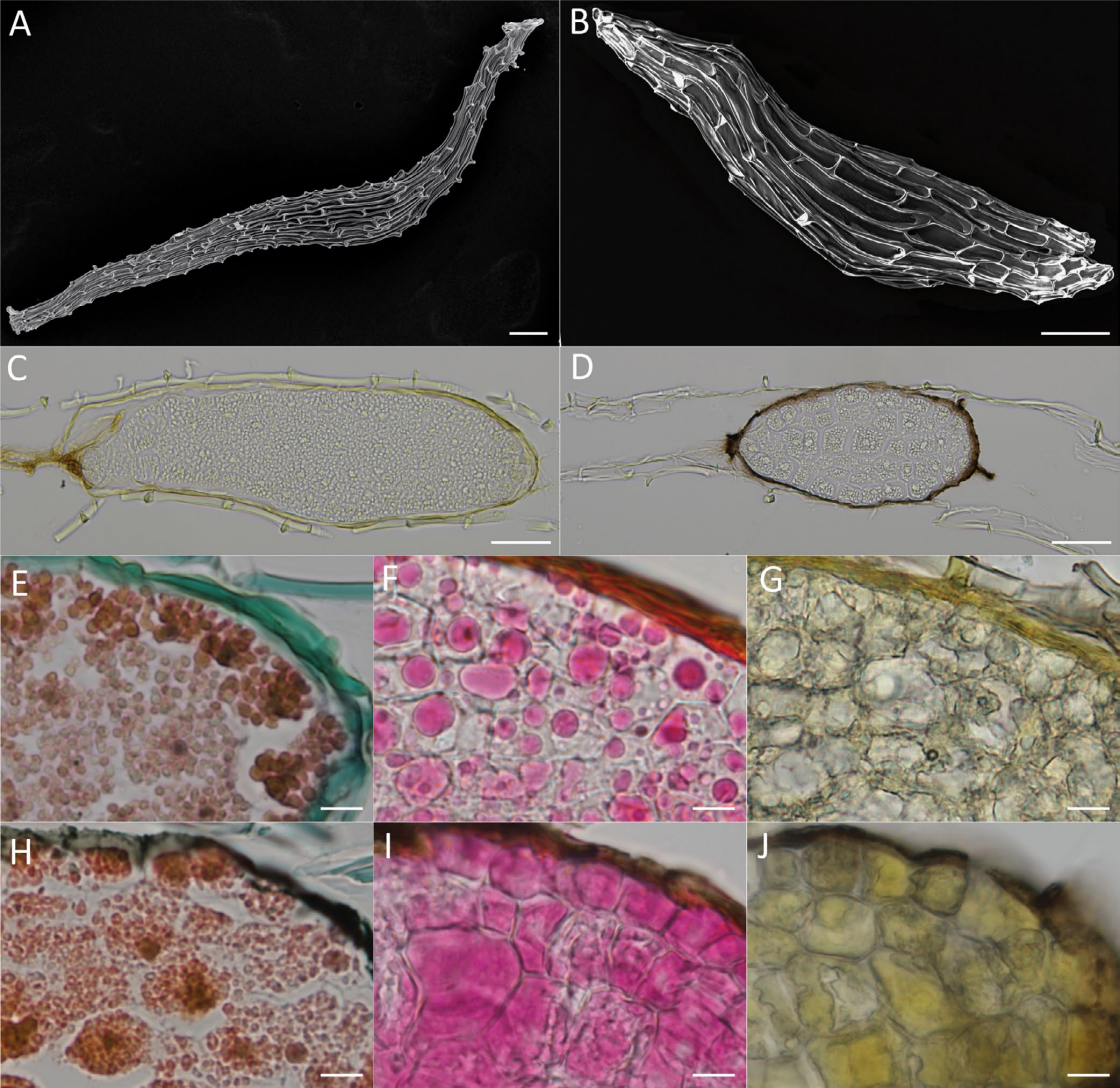
Seed anatomy of *Arundina graminifolia* and *Spathoglottis plicata* . Appearance of ripe seeds of (A) *A. graminifolia* and (B) *S. plicata* . Detail of embryos of (C) *A. graminifolia* and (D) *S. plicata* . Histochemical detection of proteins (red) in embryos of (E) *A. graminifolia* and (H) *S. plicata* . Histochemical detection of lipids (pink) in embryos of (F) *A. graminifolia* and (I) *S. plicata* . Absence of positive histochemical detection of starch (no purple color visible) in embryos of (G) *A. graminifolia* and (J) *S. plicata* . (A, B) SEM images; (C, D) paraffin sections, no staining; (E, H) paraffin sections, Ponceau 2R + Azur staining; (F, I) cryosections, Sudan Red 7B staining; (G, J) cryosections, Lugol's solution staining. Scale bars (A, B) 100 μm, (C, D) 50 μm and (E–J) 10 μm.

Mature seeds of *A. graminifolia* and *S. plicata* showed the typical structure of an orchid globular embryo without endosperm surrounded by a dead seed coat (Figure 2A–D). The size of fully developed mature seeds, including the seed coat, was 1.62 ± 0.16 mm in length and 0.16 ± 0.01 mm in width in *A. graminifolia* , and 0.81 ± 0.13 mm in length and 0.15 ± 0.02 mm in width in *S. plicata* (mean ± SE, *n* = 10).

The embryo of *A. graminifolia* was oval (Figure 2C) with a length of 0.37 ± 0.03 mm and a width of 0.12 ± 0.01 mm (mean ± SE, *n* = 10). The embryo had slightly larger cells at the micropylar end than cells at the chalazal end (Figure 2C). *S. plicata* embryos were globular, smaller (Figure 2D) with a length of 0.20 ± 0.01 mm and a width 0.10 ± 0.01 mm (mean ± SE, *n* = 10). Median weight of the *S. plicata* seed was 1.35 μg and of *A. graminifolia* 3.00 μg (*n* = 7).

### 3.2 Storage Compounds in Mature Seeds

Histochemical staining on sections showed the presence of proteins (Figure 2E,H) and lipidic substances (Figure 2F,I) in mature embryos of both species, but we did not perform their more precise quantification by biochemical methods. Histochemical detection of storage compounds on sections from mature seeds did not detect starch in the embryos of either species as we did not observe a positive reaction with Lugol's solution in any of the mature embryos examined (*n* = 10–15, Figure 2G,J). However, a small amount of starch (19.8 μg/mg and 8 μg/mg respectively) was detected in whole dry mature seeds of both species by a more sensitive HPLC analysis (Figure S3). We also detected relatively low concentrations of soluble saccharides in dry seeds. Sucrose was the most abundant sugar in both *A. graminifolia* and *S. plicata* (average 73 and 42 μg/mg) respectively, while only low amounts of fructose (average 1.6 vs. 0.9 μg/mg), glucose (average 1.4 and 1.7 μg/mg) and inositol (average 1 and 0.6 μg/mg) were detected. Dry seeds of *A. graminifolia* contained almost twice the amount of total soluble saccharides (average 77 μg/mg) and starch (average 20 μg/mg) compared to *S. plicata* (45 μg/mg soluble saccharides and 8 μg/mg of starch, Figure S3). Because this analysis was performed on entire seeds, the detected starch cannot be localized specifically to the embryo and may also originate from other seed tissues.

### 3.3 Asymbiotic Germination and Growth on Different Carbon Sources

**FIGURE 3:**
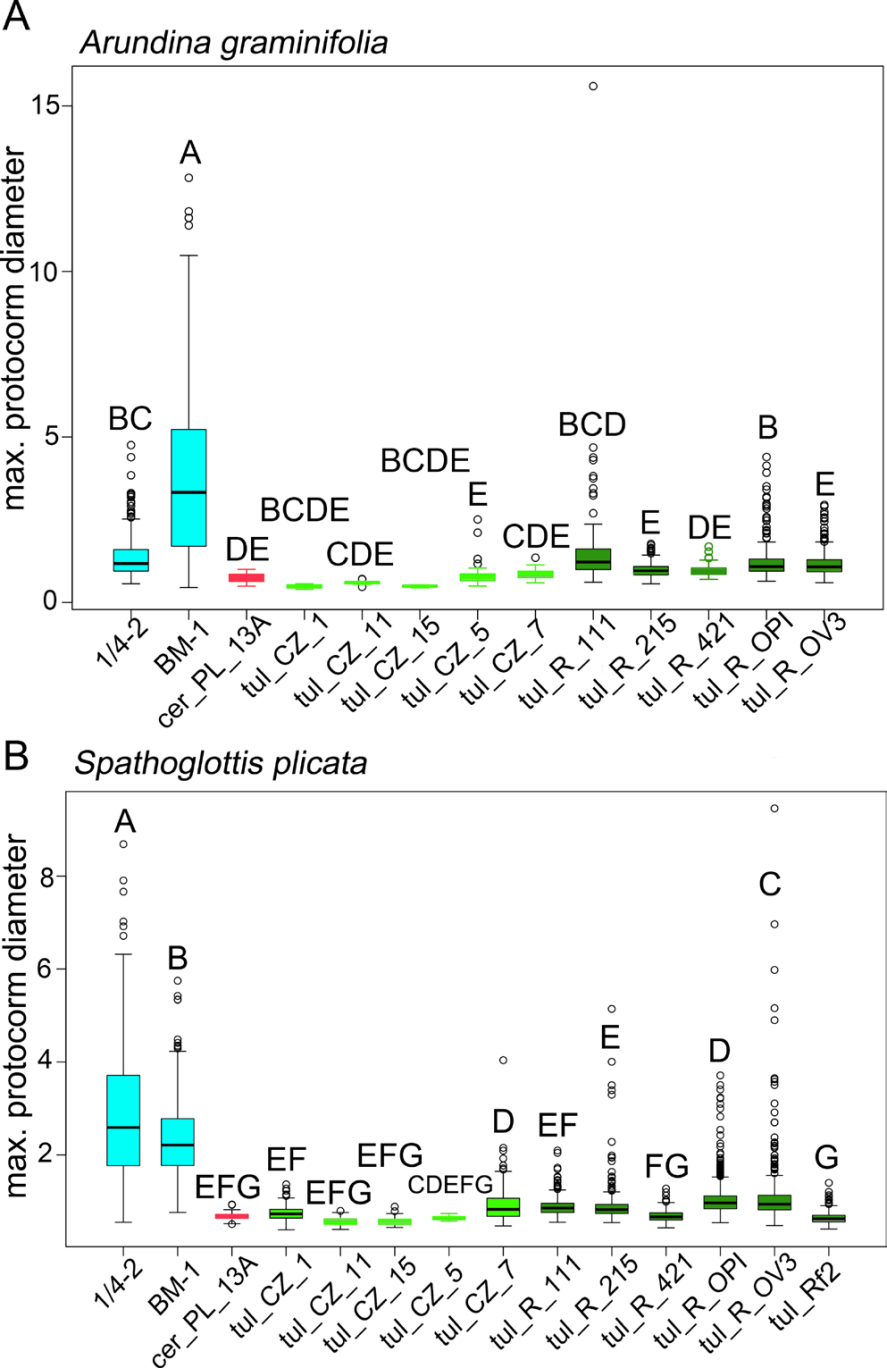
Maximum protocorm diameter in mm of (A) *Arundina graminifolia* and (B) *Spathoglottis plicata* on different experimental variants. Differences between variants were assessed by ANOVA followed by TukeyHSD test. Blue color marks asymbiotic controls, red Ceratobasidiaceae isolates, yellow Serendipitaceae, pale green European Tulasnellaceae and dark green African Tulasnellaceae.

We have successfully grown plants from seed to green plantlets, with maximum germination rates of over 95% for both orchid species. Of the two asymbiotic control media tested, ¼‐2 and BM‐1, the former performed slightly better for the cultivation of both species, although the difference was not statistically significant (Figures S1 and S2, Figure 3, Table S3).

There were significant differences in asymbiotic protocorm formation experiments with BM‐1 media containing different carbon sources, with “BMfull” followed by the “BMs” medium supplemented with sucrose only having the highest germination rate in *A. graminifolia* (Figure S4A). In the case of *S. plicata* , the “BMfull” medium and “BMs” yielded the highest protocorm formation rates too, but their difference was not significant (Figure S4B). In both species, the protocorm formation rate was lowest on medium containing amino acids and no carbon source (“BMaa” and “BM−” respectively, Figure S4).

**FIGURE 4:**
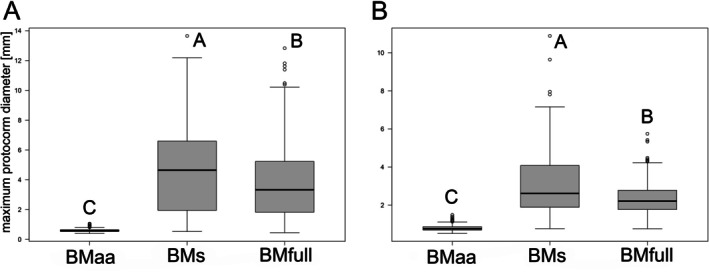
Maximum protocorm diameter in mm (protocorms with rhizoids only) of (A) *Arundina graminifolia* and (B) *Spathoglottis plicata* on media supplemented with different carbon sources. Differences between variants were assessed by ANOVA followed by TukeyHSD test. Different letters show differences between variants.

The largest protocorms and seedlings in both tested species were observed on “BMs” medium followed by “BMfull,” and the difference between these two media was significant (Figure 4, Table S3). However, seedlings and protocorms on “BMs” had almost no rhizoids while these were abundant on “BMfull” (Figures S1U,X and S2T,W). Seedlings on “BMaa” enlarged only slightly and on “BM−” did not enlarge visibly compared to imbibed seeds. No additional germination was observed 60 days after sowing. Protocorms started to form shoots between 30 and 50 days after sowing. In the first experiment, we have observed high mortality of imbibed seeds and no germination of both species on “BMaa+” medium (Figure S5). Therefore, this variant was excluded from further experiments presented here (Figure 4, Figure S4). However, due to the consistent lethality of the high amino acid concentration (BMaa+), we report these preliminary results to highlight the inhibitory effect of this variant. Seeds on all variants were imbibed (Figures S1 and S2).

### 3.4 Phylogenetic Analysis of Tulasnellaceae

Phylogenetic analysis recovered two main lineages: the basal Tulasnellaceae and the genus *Tulasnella* itself (also referred to as core Tulasnellaceae by Vogt‐Schilb et al. 2020).

**FIGURE 5:**
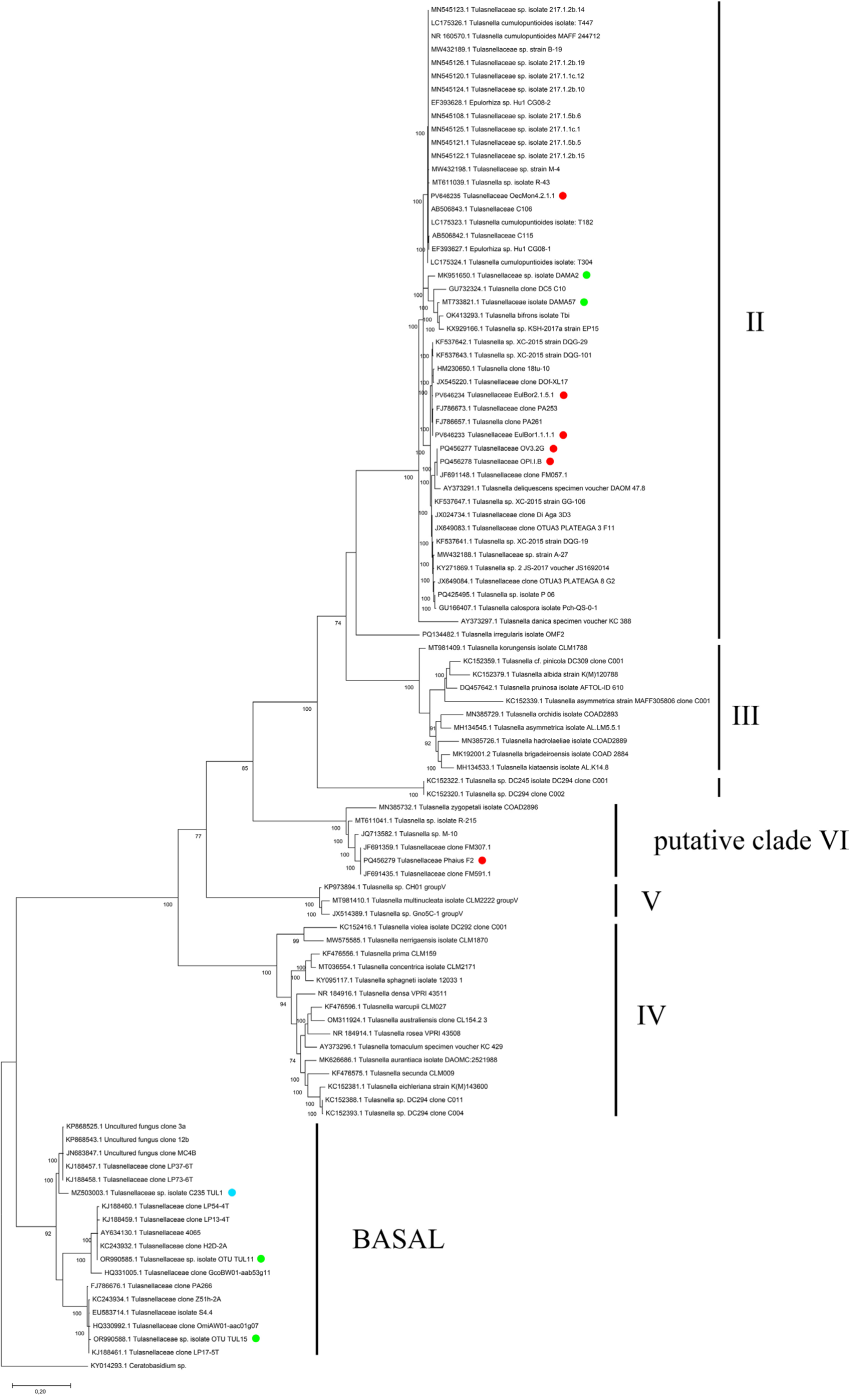
Phylogeny of Tulasnellaceae fungi using nrITS sequences. Analysis was performed by Maximum Likelihood and the tree with the highest log likelihood is shown. Numbers in the branches represent the bootstrap percentage of 1000 replicates (values above 70% are shown), dots indicate the fungi used in this study; red dot those originating from La Réunion, blue from France and green from Czechia.

Within core Tulasnellaceae, most lineages corresponded to clades previously recognized by Arifin et al. (2022). In addition, our analysis recovered one well‐supported lineage that did not clearly correspond to previously recognized clades. For convenience, we refer to this lineage informally as putative clade VI. This designation is used only within the context of the present phylogenetic analysis and does not represent a formal taxonomic description or diagnostic delimitation. The sequences analyzed in this study belonged to basal Tulasnellaceae, clade II, and putative clade VI (Figure 5).

### 3.5 Symbiotic Germination and Growth With Fungi From Different Regions

**FIGURE 6:**
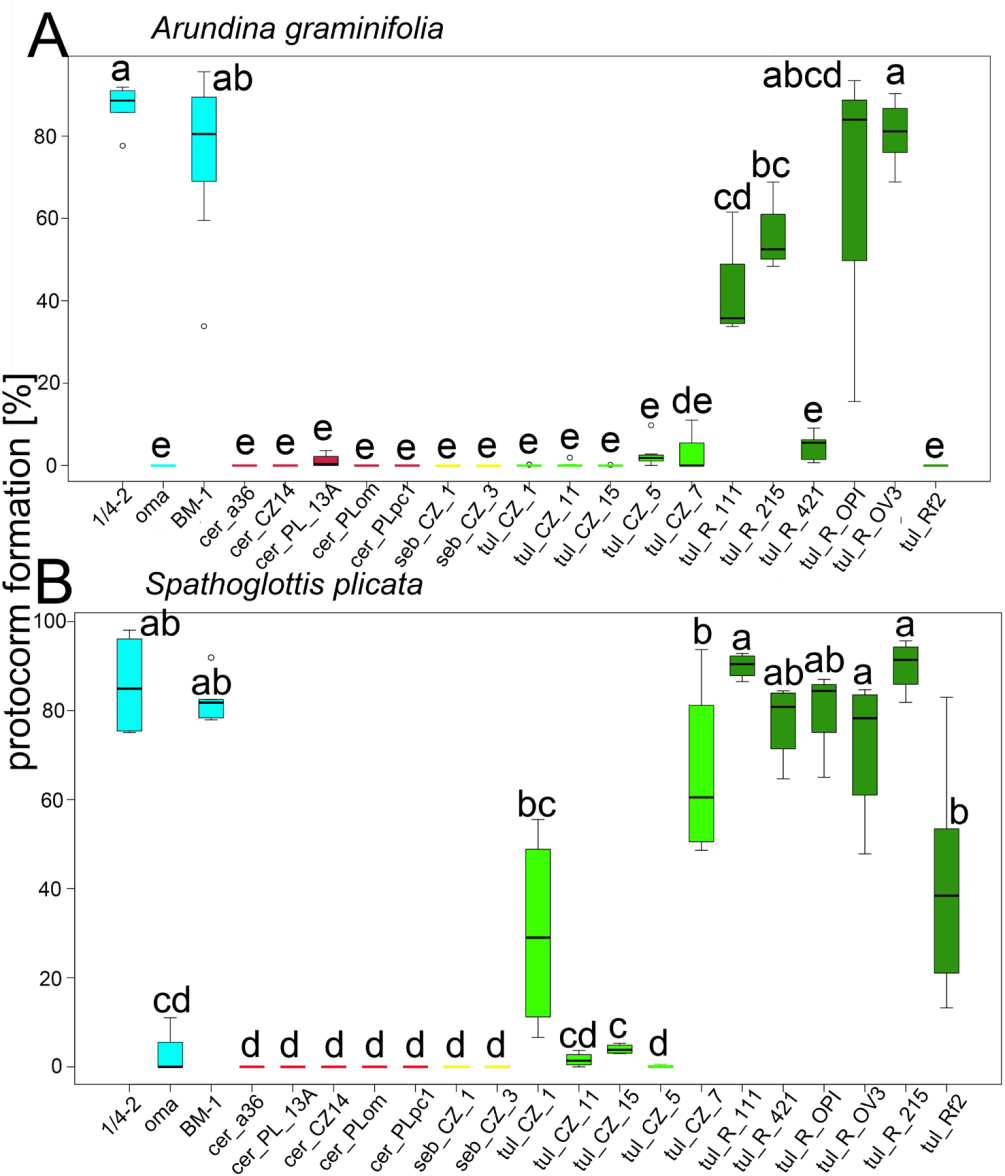
Percentage of protocorm formation in (A) *Arundina graminifolia* and (B) *Spathoglottis plicata* on different fungal isolates. Different letters indicate significant differences among variants according to pairwise Wilcoxon tests. Blue indicates asymbiotic controls; red, Ceratobasidiaceae isolates; yellow, Serendipitaceae; light green, European Tulasnellaceae; and dark green, La Réunion.

OMA without an inoculated fungus did not support further growth of the orchids and promoted only low germination rates with a few very small protocorms of *S. plicata* , possibly using seed reserves and a small amount of soluble organic compounds in the medium (Figures S1 and S2, Figure 6). There were significant differences in germination and protocorm formation trates and size of protocorms between fungal treatments in both species (*p* < 0.05, Table S3). *A. graminifolia* and *S. plicata* both germinated with all Tulasnellaceae (~35%–90%), except *A. graminifolia* with isolate tul_Rf2 (0%; Figure S6). However, protocorm formation of *A. graminifolia* with basal Tulasnellaceae (tul_CZ1, tul_CZ11, and tul_CZ15) and with tul_CZ5 from clade II did not significantly differ from non‐supportive isolates or controls without fungus (oma), with protocorm formation rates were below 10%, although some individual protocorms were observed with tul_CZ5 (Figure 6A). Similarly, very low rates of protocorm formation in *S. plicata* , also below 10%, were observed with isolates tul_CZ5 (clade II), tul_CZ11, and tul_CZ15 (basal Tulasnellaceae) not differ from the oma control (Figure 6B). Interestingly, *S. plicata* formed protocorms with tul_CZ1 from basal Tulasnellaceae (~30%). Isolates of *Tulasnella* isolated from tropical orchids in La Réunion generally supported protocorm formation better (40%–90% rate) than those isolated from temperate orchids in Europe, with the exception of tropical isolate tul_Rf2 (putative clade VI) which supported only *S. plicata* and European isolate tul_CZ7 (clade II) which allowed similar protocorm formation rates as tropical isolates of *Tulasnella* (Figure 6B). Further growth of the *A. graminifolia* protocorms was observed only with isolates from La Réunion, with the exception of tul_CZ5 (clade II). Isolates from La Réunion also formed bigger protocorms (Figure 3, Figures S1 and S2). Seeds of both species generally failed to form protocorms with Ceratobasidiaceae or Serendipitaceae isolates, with one weak exception in *A. graminifolia* with Ceratobasidium PL13a. However, this *Ceratobasidium* only allowed the formation of very few and small protocorms (Figure 3, Figures S1D and S2D). In addition, testa rupture but no protocorm formation was also observed with *Serendipita* seb_CZ3 and *Ceratobasidium* PLpc1 (Figure S6A). In general, *A. graminifolia* seeds ruptured the testa in response to a larger number of fungal isolates than those that ultimately supported formation of protocorms with rhizoids (Figure S6).

### 3.6 Symbiotic Germination of *A. graminifolia* Seeds From Different Regions

**FIGURE 7:**
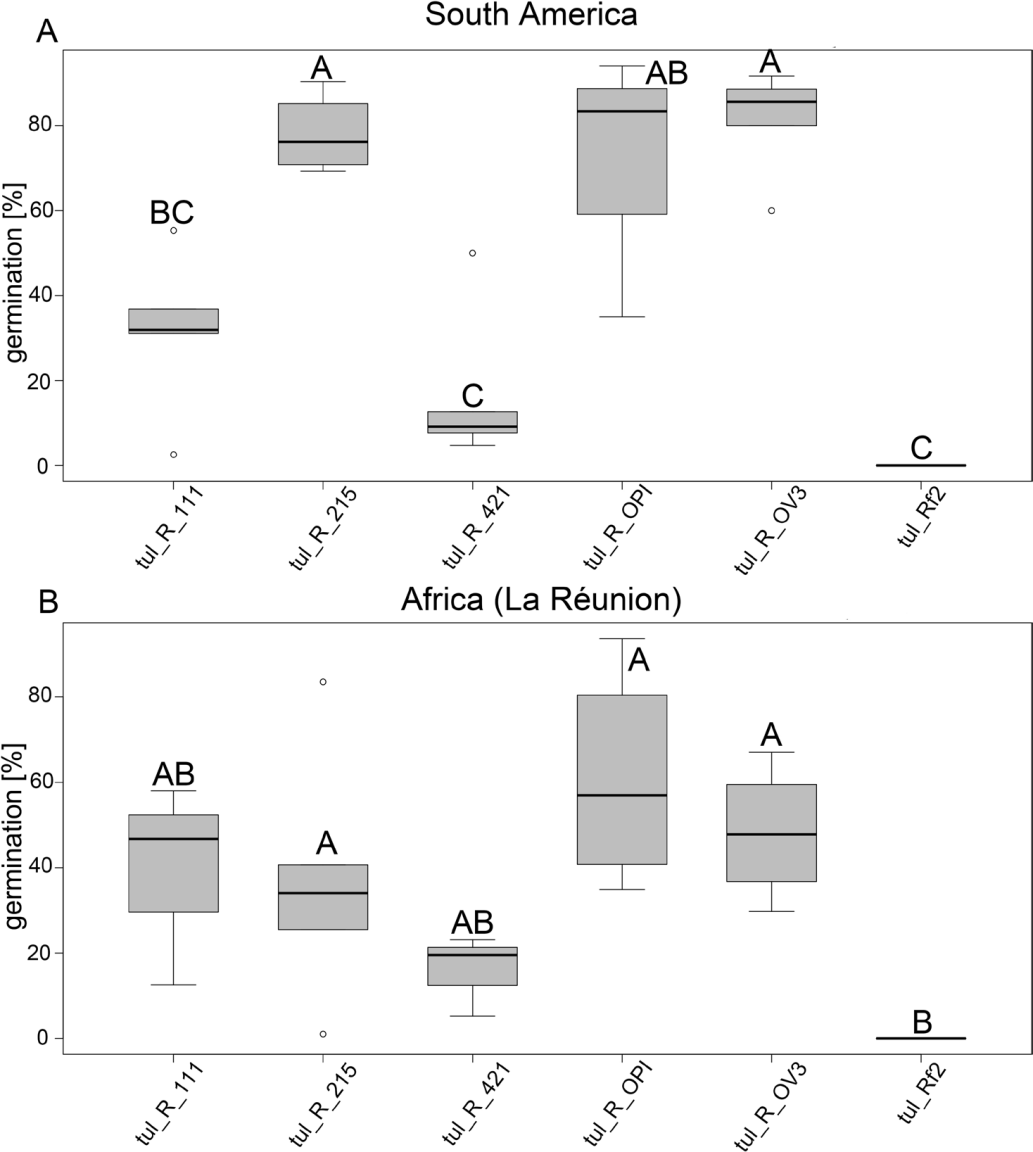
Germination percentage of *Arundina graminifolia* seeds originating from Colombia, South America (A) and La Réunion (B) on tropical Tulasnella isolates from La Réunion (Africa). Different letters show differences between variants according to Tukey HSD test.

This experiment was evaluated earlier, 60 days after sowing, because contaminations prevented longer incubation (all other experiments were repeated and counted 90 days after sowing). Similar germination percentages of seeds originating from Colombia and La Réunion were observed (Figure 7, Table S3). Out of six isolates from La Réunion, five belonged to clade II and one isolate, tul_R_215, belonged to putative clade VI. The five clade II isolates supported germination to different extents, whereas tul_Rf2 did not support germination in either seed origin. Among the supportive isolates, tul_R_OV3 and tul_R_OPI resulted in the highest germination percentages. Tul_R_215 was the only isolate that showed different germination responses (approximately half of seeds germinated) in *A. graminifolia* seeds from the two geographical origins; however, this difference was not statistically significant.

## 4 Discussion

We found that one factor that may contribute to their success outside their natural ranges is their ability to colonize a wide array of mycorrhizal fungi from the Tulasnellaceae family (this study; Aewsakul et al. 2013; Athipunyakom et al. 2004; Minea et al. 2004; Pereira Angeli 2019). However, other factors are also likely to contribute, namely seed size, which likely offers greater available resources, and—as previously suggested—the high number of seeds resulting from successful pollination and the ability to thrive in ruderal habitats (Ackerman et al. 2014). *Spathoglottis plicata* appears to have a higher potential to establish mycorrhizal associations with new fungal isolates for both germination and further growth (this study, Aewsakul et al. 2013; Athipunyakom et al. 2004; Minea et al. 2004), while *A. graminifolia* produces large embryos, indicating a relatively higher total reserve available for germination, thus may lower the time of full dependency on fungus and possibly higher invasive success.

### 4.1 Broad Compatibility With Tulasnellaceae May Facilitate Establishment Outside the Native Range

Both studied species were germinating with isolates belonging to Tulasnellaceae (Aewsakul et al. 2013; Ma et al. 2003; Pereira Angeli 2019; Shan et al. 2002), also when those isolates originated outside their native range (Pereira Angeli 2019). In the roots of both species, other “rhizoctonias” (Athipunyakom et al. 2004; Pereira Angeli 2019) and even non‐rhizoctonias (Meng et al. 2019) have previously been reported from roots of these orchids, but did not support germination. Similarly, in this study, only Tulasnellaceae supported protocorm formation, with the exception of one *Ceratobasidium* isolate in *A. graminifolia* showing a very low number and size of protocorms. Fungal isolates allowing protocorm formation of *S. plicata* belonged to clade II and putative clade VI of core Tulasnella and to the basal clade, while those supporting *A. graminifolia* only to clade II of core Tulasnella. Notably, protocorm formation but to a lower extent also germination of both species was higher with local tropical fungal isolates belonging to a single phylogenetic group (clade II). This was more pronounced in *A. graminifolia* , having very low germination percentages and lower protocorm formation rates with fungi which originated from Europe and those from other phylogenetic groups. However, European isolates “tul_CZ5” and “tul_CZ7” supported the formation of big protocorms in *A. graminifolia* , while more European isolates supported germination and protocorm growth of *S. plicata* , comparable to native isolates.

Other invasive orchids *Microtis media* or *Disa bracteata* have been found to associate with a very broad range of mycorrhizal fungi (De Long et al. 2013; Bonnardeaux et al. 2007), and similarly *Oeceoclades maculata* is also a generalist toward mycorrhizal fungi when adult, but more specific during germination (Bayman et al. 2016). Similar to the study of Bonnardeaux et al. (2007) on *Disa bracteata*, some of our fungal isolates which supported seed germination of both species in our study originated as far as 9000–10,000 km apart. Moreover, in this study, seeds from Colombia had a similar preference toward fungi from La Réunion as local seeds from La Réunion. This indicates that both species are effective colonizers capable of forming symbiotic relationships with fungi from other continents while maintaining a consistent preference toward Tulasnellaceae.

### 4.2 High Seed Production and Germination

Whether species become invasive or not can be influenced by propagule pressure (e.g., Richardson and Pyšek 2006; Von Holle and Simberloff 2005). Orchids are well known for their ability to produce massive amounts of seeds (Arditti and Ghani 2000) and already Darwin (1859) noted that orchids have tremendous colonization potential because of their high seed production. Both species in this study are no exception and on top of that they also reached germination above 95% consistent with other studies on *S. plicata* (Minea et al. 2004). However, high number of seeds is a general feature of orchids and high germination percentages are often observed even among noninvasive orchid species (Bonnardeaux et al. 2007; Figura et al. 2020; Ponert et al. 2011). Thus, while high seed production and germination are consistent with Darwin's early observations and are commonly found in orchids, these traits alone do not explain the invasive success of *A. graminifolia* and *S. plicata* .

Moreover, while collecting seeds in situ, we observed relatively high pollination success of *A. graminifolia* and complete fruit set in *S. plicata* , which is capable of self‐pollination (Minea et al. 2004). Seeds with developed embryos were present in high numbers in both species. However, the pollination strategy of these two species differs slightly (Ackerman et al. 2014; Sugiura 2014). Both species are pollinated, among others, by widespread *Xylocopa* spp. bees, which are available in both native and newly colonized habitats (Ackerman et al. 2014; Sugiura 2014). Despite being self‐compatible, *A. graminifolia* is incapable of self‐pollination. This species is more generalistic with respect to pollinators (including widespread bees *Apis mellifera* ), despite being nectarless (Ackerman et al. 2014; Sugiura 2014). However, a study (Ackerman et al. 2024) showed variable reproductive success among different novel island habitats, probably reflecting the available pollinator pool.

The high number of well‐developed seeds with high germination percentages observed in this study likely contributes to the success of these species as colonizers and invaders.

### 4.3 Embryo Size Matters

Orchid seeds show a remarkable variation in length, typically ranging from 0.05–2 mm (rarely even 6 mm) as well as in width (0.01–0.9 mm). Such variations may impact germination success, even within a genus or a species (Arditti and Ghani 2000; Rasmussen 1995). Both studied species produce relatively large seeds compared to most orchids (Arditti and Ghani 2000; Barthlott et al. 2014), which may be a feature shared by some invasive orchids (Riverón‐Giró et al. 2017). Different sizes of *A. graminifolia* seeds were reported ranging from 0.781 × 0.082 mm (length × width; Verma et al. 2012) to 1.3 × 0.15 mm (length × width); (Nishimura 1990). In our study we measured even bigger lengths of *A. graminifolia* (1.62 ± 0.16 mm) and widths (0.16 ± 0.01 mm) in non‐native areas. However, we measured only well‐developed mature seeds. Seeds of *S. plicata* are reported to be a bit smaller with length 0.8 mm and widths 0.2 mm according to (Minea et al. 2004). This is congruent with our measurements (0.81 × 0.15 mm) of *S. plicata* .

However, since the air‐filled testa of orchid seeds is disproportionately large relative to the embryo, it is more meaningful to compare embryo size. In general, orchid embryo sizes range from 0.08 × 0.06 mm to 0.32 × 0.2 mm (length × width) (Arditti and Ghani 2000). Reported embryo sizes of *A. graminifolia* also vary ranging from 0.238 × 0.076 mm (length × width; Verma et al. 2012) to 0.4 × 0.15 mm (length × width; Nishimura 1990). Our results showed similar embryo sizes (0.37 × 0.12 mm, mean ± SE, *n* = 10) to those reported by Nishimura (1990). Concerning embryos of *S. plicata* , length (0.26 mm), width (0.14 mm) and a volume of 5.69 mm^3^ reported by Arditti and Ghani (2000) are congruent with our measurements (0.20 × 0.10 mm, mean ± SE, *n* = 10). Following the categorization of orchid seed sizes by Barthlott et al. (2014), both studied species would fall into the second largest category named “large seeds,” with *A. graminifolia* reaching the upper boundary of this category (900–2000 μm). The seed weight could also be indicative, ranging from 0.3 μg to 26 μg within Orchidaceae (Arditti and Ghani 2000). Despite the fact that seed weight of only a few orchid species has been measured so far, based on Arditti and Ghani (2000), we estimated the average (4.5 μg) and median (3 μg) weight of an orchid seed. *A. graminifolia* seed weight (3 and 2.9 μg) is therefore around average and median. *S. plicata* is way lighter with both average and median weight around 1.1 μg. However, in other parameters—length and width of the whole seed and also of embryo, both studied orchids, but especially *A. graminifolia* are among the largest reported in Orchidaceae.

### 4.4 Typical Orchid Embryos and Amount of Reserves

The majority of initially mycoheterotrophic plants have very small seeds, known as dust seeds (Eriksson and Kainulainen 2011; Figura et al. 2019). Orchids are no exception, with embryogenesis ceased in the globular stage and lack of endosperm in mature seeds (Yeung 2017). Despite orchid embryos usually showing no signs of histodifferentiation, some studies reported a gradient of cell sizes within the embryo (Lee and Yeung 2018) or even the formation of a rudiment of cotyledon (Nishimura 1991; Vinogradova and Andronova 2002). In the case of *A. graminifolia* , we observed larger cells at the micropylar part of the embryo corresponding to the previous findings (Nishimura 1991). However, we consider embryos of both species as typical orchid embryos with development ceased at the globular stage without cotyledons.

Orchid seeds are commonly referred to as reserveless. However, this is incorrect, as the general consensus is that they contain considerable amounts of protein and lipid reserves. Soluble saccharides are reported only rarely (Manning and Van 1987), and the same is true for starch (Shun‐xing and Jin‐tang 1990). Indeed, we detected only protein and lipid reserves by histochemical tests on sections of mature seeds in both species. Although we have not quantified them exactly, the intensity of histochemical staining was similar in both species and it is very likely that the seeds of *A. graminifolia* may contain a greater total amount of reserves because the seeds are approximately twice as large. HPLC analysis showed low but not negligible amounts of soluble saccharides (mainly sucrose) and starch, with roughly double amounts in *A. graminifolia* . Their amounts seemed comparable to other orchid species, but a more precise quantification is required to accurately compare interspecific variation in the content of storage compounds in orchids. In addition, intraspecific differences among samples should be interpreted cautiously, as they likely reflect both biological variation, particularly differences in proportion of fully developed seeds, and methodological variation associated with the small dry mass of some samples. Given the limited number of introduced‐range populations sampled and the small number of HPLC samples analyzed per species, maternal effects, population‐level variation, and seed maturation conditions may have influenced the measured reserve contents and germination responses.

Therefore, despite some gradient of cell sizes in *A. graminifolia* , embryo development is typical for orchids; however, larger embryo size and higher seed weight allow it to bear slightly more reserves compared to other orchids—this is more pronounced in *A. graminifolia* .

### 4.5 Carbon Source for Germination

Cultivation medium containing both sucrose and amino acids (“BMfull”; glutamine and glycine) yielded the highest germination percentages and produced bigger protocorms in both species but especially in *A. graminifolia* . *S. plicata* germinated almost equally well on medium with only sucrose (“BMs”). This is consistent with limited research suggesting that amino acids (Cameron et al. 2008; Fochi et al. 2017) and, probably to a greater extent, saccharides (Li et al. 2022; Ponert et al. 2021) contribute to the transfer of carbon from fungi to orchids. Sucrose is the preferred saccharide for many orchid species as it can be utilized easily (Ponert et al. 2011, 2021; Ponert and Lipavská 2017). However, further growth of both species was slightly better on “BMs” medium containing only sucrose. Both species formed green shoots 30–50 days after sowing, by this time possibly already obtaining carbon from photosynthesis. From in vitro cultures, it is generally known that even adult autotrophic plants can benefit from externally added carbon in the cultivation media, although they are not dependent on it in natural conditions. Carbohydrates in the culture medium supply energy and carbon for growth and in vitro plants are commonly growing heterotrophically even when they are green (reviewed by Yaseen et al. 2013).

Germination on “BMaa” medium containing only amino acids and no sucrose was significantly lower than on sucrose‐containing medium suggesting that amino acids are a poor carbon source, at least during the germination phase in vitro. The exclusion of amino acids from media reduced germination in *A. graminifolia* but promoted growth of already germinated protocorms of both species. In addition, the high content of amino acids (BMaa+) caused the seeds to darken; therefore, adding high levels of amino acids was not beneficial, but lethal. Both species were not able to form protocorms without externally added carbon (“BM−”), which supports the current understanding that no orchid is currently known to have escaped initial mycoheterotrophy and can germinate without external carbon (Rasmussen 1995).

### 4.6 Potential Ecological Impacts of Orchid Invasions

Biotic interactions are complex and newly introduced species may acquire novel interactions, including beneficial mutualisms, that facilitate naturalization and invasion (Richardson et al. 2000; Simberloff and Von Holle 1999). Both *A. graminifolia* and *S. plicata* have been identified as posing a significant threat to the Hawaii flora, with a rating of “High risk” as outlined in the Hawaii Pacific Weed Risk Assessment (Clifford and Kobayashi 2012). The ability of both species to colonize new areas is remarkable, underlined by the observation of *A. graminifolia* being one of the first colonizers of Krakatau after its explosion (Partomihardjo 2003). Similarly, in this study, we observed *S. plicata* colonizing lava fields and primary rainforest, even in locations far from human settlements. Combined with our findings on high germination percentages, efficient protocorm formation, and the ability to form associations with a wide range of *Tulasnellaceae* species—including those from distant phylogenetic lineages and continents—these results suggest that *S. plicata* , in particular, may pose a risk to newly invaded habitats. *A. graminifolia* seems to spread less in novel areas. Nevertheless, both species exhibit traits typical of weedy plants allowing them to compete with native flora for abiotic niches and biotic interactions.

## 5 Conclusions

We conclude that the success of both species, and especially of *S. plicata* , may be promoted by their ability to form protocorms with a broad range of *Tulasnellaceae* isolates, including those isolated from distant continents. This is especially the case of *S. plicata* , which can also associate with phylogenetically distant clades within the Tulasnellaceae. In contrast, *A. graminifolia* displays a lower degree of generalism toward foreign Tulasnellaceae and requires symbionts from a single clade. However, *A. graminifolia* has slightly larger and heavier seeds with embryos among the largest in the Orchidaceae family, possibly compensating for its lower fungal generalism. Nevertheless, as shown in previous studies, both species exhibit a high degree of ecological generalism in terms of habitat preference and pollinator interactions, with *S. plicata* also capable of successful self‐pollination.

We demonstrate that both species possess typical orchid embryos that remain at the globular stage of embryogenesis. Moreover, both have relatively large embryos by orchid standards, and those of *A. graminifolia* appear to contain more storage compounds. Overall, our study sheds light on the diverse mechanisms that may underlie orchid invasions, as illustrated by two species with contrasting strategies and invasion trajectories.

## Author Contributions

**Tomáš Figura:** conceptualization (lead), data curation (equal), funding acquisition (lead), investigation (equal), methodology (equal), project administration (equal), resources (equal), supervision (equal), visualization (equal), writing – original draft (lead), writing – review and editing (equal). **Edita Tylová:** data curation (equal), formal analysis (equal), investigation (equal), methodology (equal), visualization (equal), writing – original draft (equal), writing – review and editing (supporting). **František Novák:** data curation (supporting), investigation (equal), visualization (equal), writing – review and editing (supporting). **Vincent S. F. T. Merckx:** supervision (equal), writing – review and editing (equal). **Jan Ponert:** formal analysis (equal), investigation (equal), writing – review and editing (supporting). **Julita Minasiewicz:** data curation (equal), investigation (equal), writing – review and editing (supporting). **Marc‐André Selosse:** supervision (lead), writing – review and editing (equal). **Florent Martos:** conceptualization (equal), formal analysis (equal), investigation (equal), methodology (equal), writing – review and editing (equal).

## Funding

This work was supported by H2020 European Research Council, 101045057. Grantová Agentura České Republiky, 23‐05310O. Akademie Věd České Republiky, RVO 6798593.

## Conflicts of Interest

The authors declare no conflicts of interest.

## Supporting information


**Figure S1:** Seeds and protocorms of *Arundina graminifolia* in all asymbiotic (A, B, U, V, W, X) and symbiotic (C–T) experimental variants tested (A) ¼‐2, (B) oma, (C) cer_a36, (D) cer_PL13, (E) cer_CZ14, (F) cer_PLom, (G) cer_pc1, (H) seb_CZ1, (I) seb_CZ3, (J) tul_CZ1, (K) tul_CZ11, (L) tul_CZ5, (M) tul_CZ15, (N) tul_CZ7, (O) tul_R_111, (P) tul_R_215, (Q) tul_R_411, (R) tul_R_OPI, (S) tul_R_OV3, (T) tul_Rf2, (U) BMs, (V) BM−, (W) BMaa, (X) full. Each photo A–X is 1 × 1 cm big.
**Figure S2:** Seeds and protocorms of *Spathoglottis plicata* (A) ¼‐2, (B) oma, (C) cer_a36, (D) cer_PL13, (E) cer_CZ14, (F) cer_PLom, (G) seb_CZ1, (H) seb_CZ3, (I) tul_CZ1, (J) tul_CZ11, (K) tul_CZl5, (L) tul_CZ15, (M) tul_CZ7, (N) tul_R_111, (O) tul_R_215, (P) tul_R_411, (Q) tul_R_OPI, (R) tul_R_OV3, (S) tul_Rf2, (T) BMs, (U) BM−, (V) BMaa, (W) BMfull. Each photo A–W is 1 × 1 cm big.
**Figure S3:** Differences in endogenous soluble saccharides and starch contents in μg/mg dry weight (A) and in μg per one seed (B) in *A. graminifolia* (samples 1, 2) and *S. plicata* (samples 3, 4).
**Figure S4:** Protocorm formation percentage (protocorms with rhizoids only) of (A) *Arundina graminifolia* and (B) *Spathoglottis plicata* on different carbon sources. Differences between variants were assessed by ANOVA followed by TukeyHSD test. Different letters show differences between variants. *p*‐value for (A) *Arundina graminifolia* was *p* = 0.0005021 and for (B) *Spathoglottis plicata p* = 0.002646.
**Figure S5:** Imbibed dead seeds of (A) *Arundina graminifolia* and (B) *Spathoglottis plicata* on medium with elevated amino acid level “BMaa+”. Scale bar 1 cm.
**Figure S6:** Percentage of (A) *Arundina graminifolia* and (B) *Spathoglottis plicata* seeds breaking testa (including protocorms) on different fungal isolates. Different letters show differences between variants according to the pairwise Wilcoxon test. Blue marked are asymbiotic controls, red are Ceratobasidiaceae isolates, yellow Serendipitaceae, pale green European Tulasnellaceae and dark green African Tulasnellaceae. *p*‐values for (A) *Arundina graminifolia* was *p* = 9.639^e−11^ and (B) *Spathoglottis plicata p* = 1.139^e−07^.

**Table S1:** Composition of cultivation media used in this study. All weights are in mg L^−1^ except of pineapple juice volume which is in mL L^−1^.
**Table S2:** Fungal strains used in this study. Taxonomic placement, isolate origin and GenBank accession number.
**Table S3:** Summary of statistical analyses of all germination and maximum protocorm diameter tests. Statistical test used, *p*‐values of statistical test for each experiment separately. KW stands for Kruskal–Wallis test, ANOVA for analysis of variance.

## Data Availability

All raw data supporting the results of this study are provided in the Supporting Information file, which includes both raw data (DATA1–DATA5) and summary tables. A dedicated metadata sheet explains the content of each sheet and defines all variables, units, and abbreviations used in the dataset.
