# Supplementary material for: Broad Fungal Compatibility and Seed Size May Facilitate Invasiveness in Two Asian Terrestrial Orchids Spathoglottis plicata and Arundina graminifolia
Source: Ecol Evol. 2026 Jun 10;16(6):e73805. doi: 10.1002/ece3.73805 (PMC13253047; doi:10.1002/ece3.73805)

Fig S1: Seeds and protocorms of *Arundina graminifolia* in all asymbiotic (A,B,U,V,W,X) and symbiotic (C-T) experimental variants tested A) ¼-2, B) oma, C) cer_a36, D) cer_PL13, E) cer_CZ14, F) cer_PLom, G) cer_pc1, H) seb_CZ1, I) seb_CZ3, J) tul_CZ1, K) tul_CZ11, L) tul_CZ5, M) tul_CZ15, N) tul_CZ7, O) tul_R_111,P) tul_R_215, Q) tul_R_411, R) tul_R_OPI, S) tul_R_OV3, T) tul_Rf2, U) BMs, V) BM-, W) BMaa, X) BMfull. Each photo A-X is 1x1cm big.
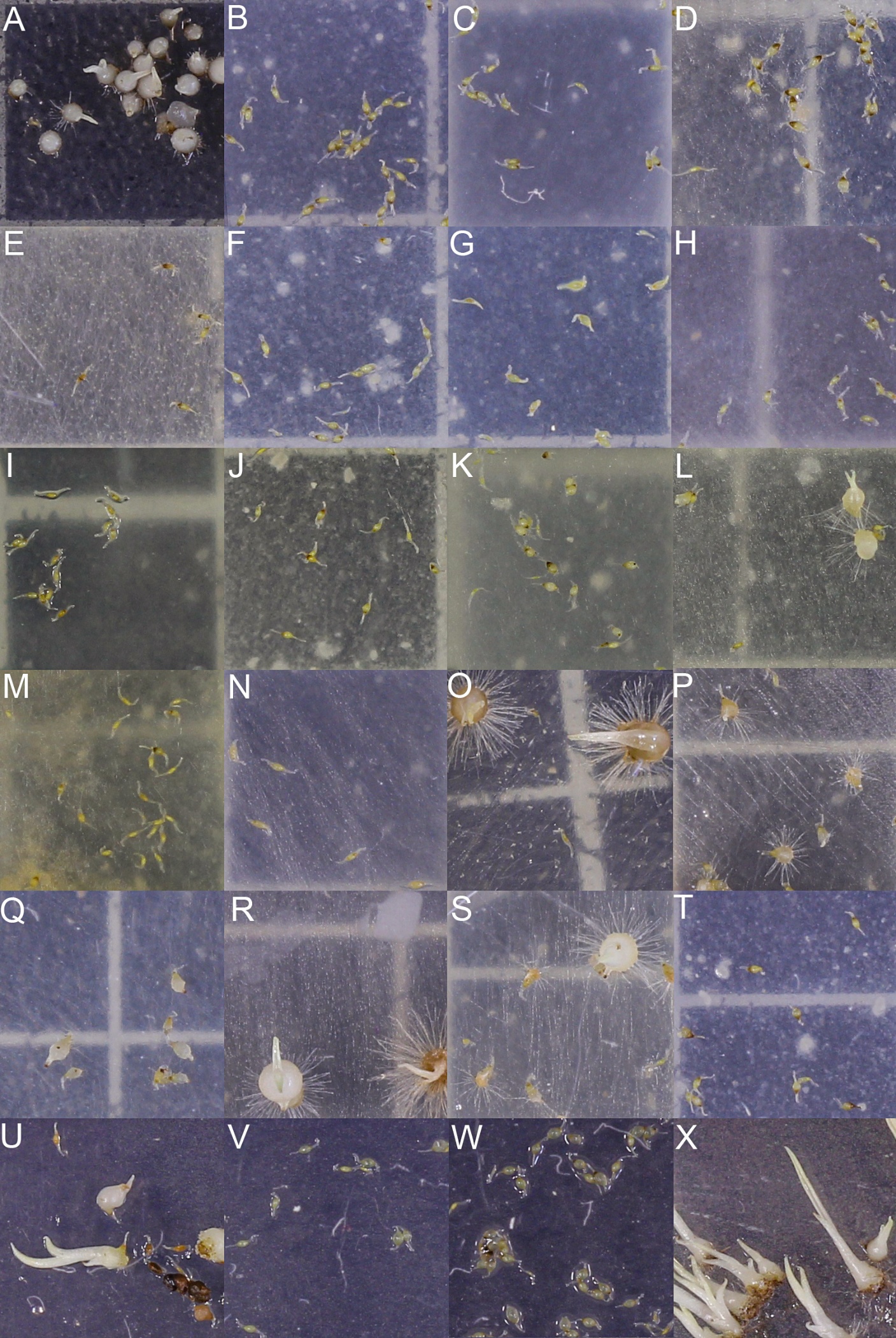


Figure S2: Seeds and protocorms of *Spathoglottis plicata* A) ¼-2, B) oma, C)cer_a36, D)cer_PL13, E) cer_CZ14, F)cer_PLom, G) seb_CZ1, H) seb_CZ3, I) tul_CZ1, J) tul_CZ11, K) tu_CZ5, L) tul_CZ15, M) tul_CZ7, N) tul_R_111,O) tul_R_215, P)tul_R_411, Q) tul_R_OPI, R) tul_R_OV3, S) tul_Rf2, T) BMs, U) BM- ,V) BMaa, W) BMfull. Each photo A-W is 1x1cm big.
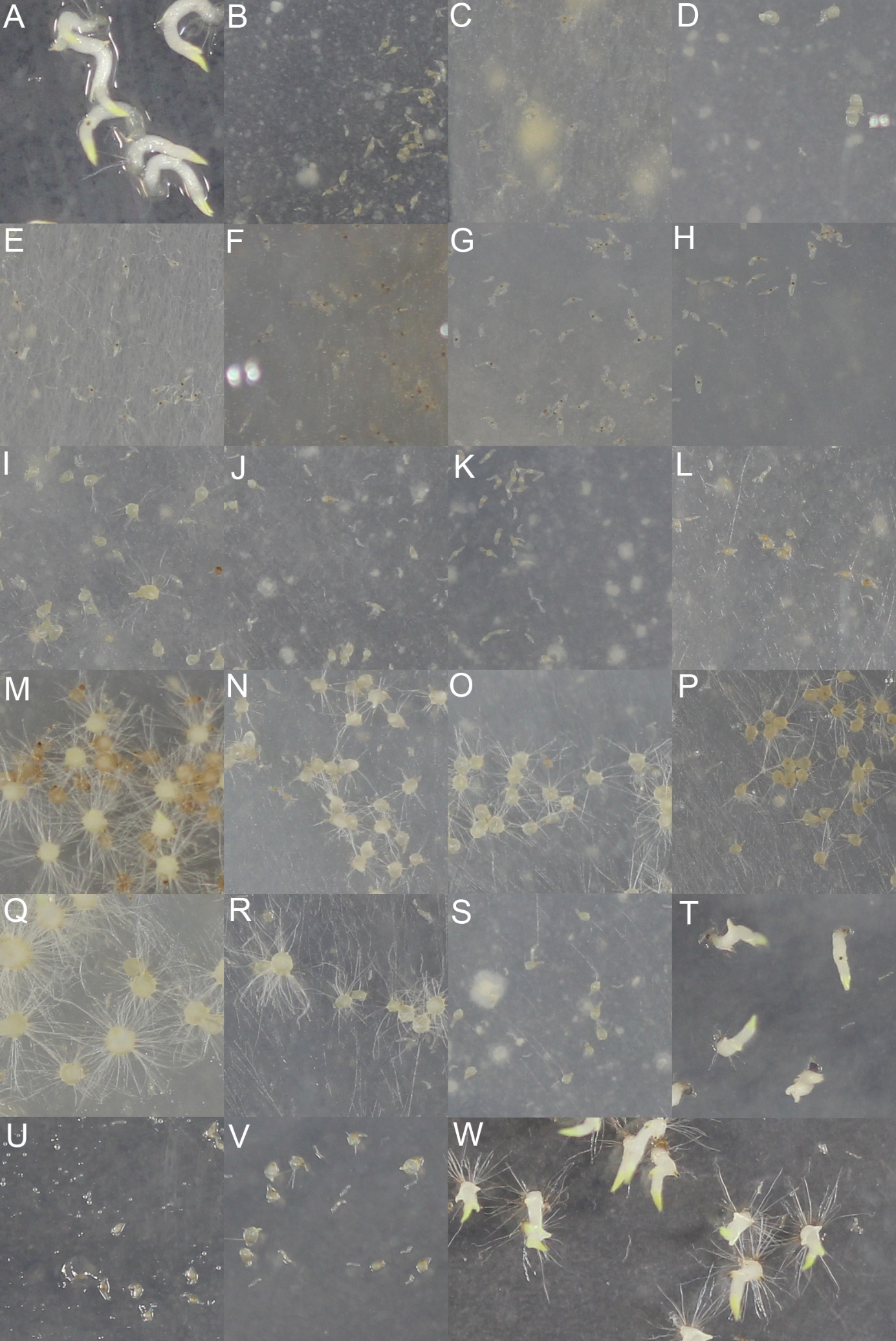


Figure S3: Differences in endogenous soluble saccharides and starch contents in µg/mg dry weight (A) and in µg per one seed (B) in *A. graminifolia* (samples 1, 2) and *S. plicata* (samples 3, 4).


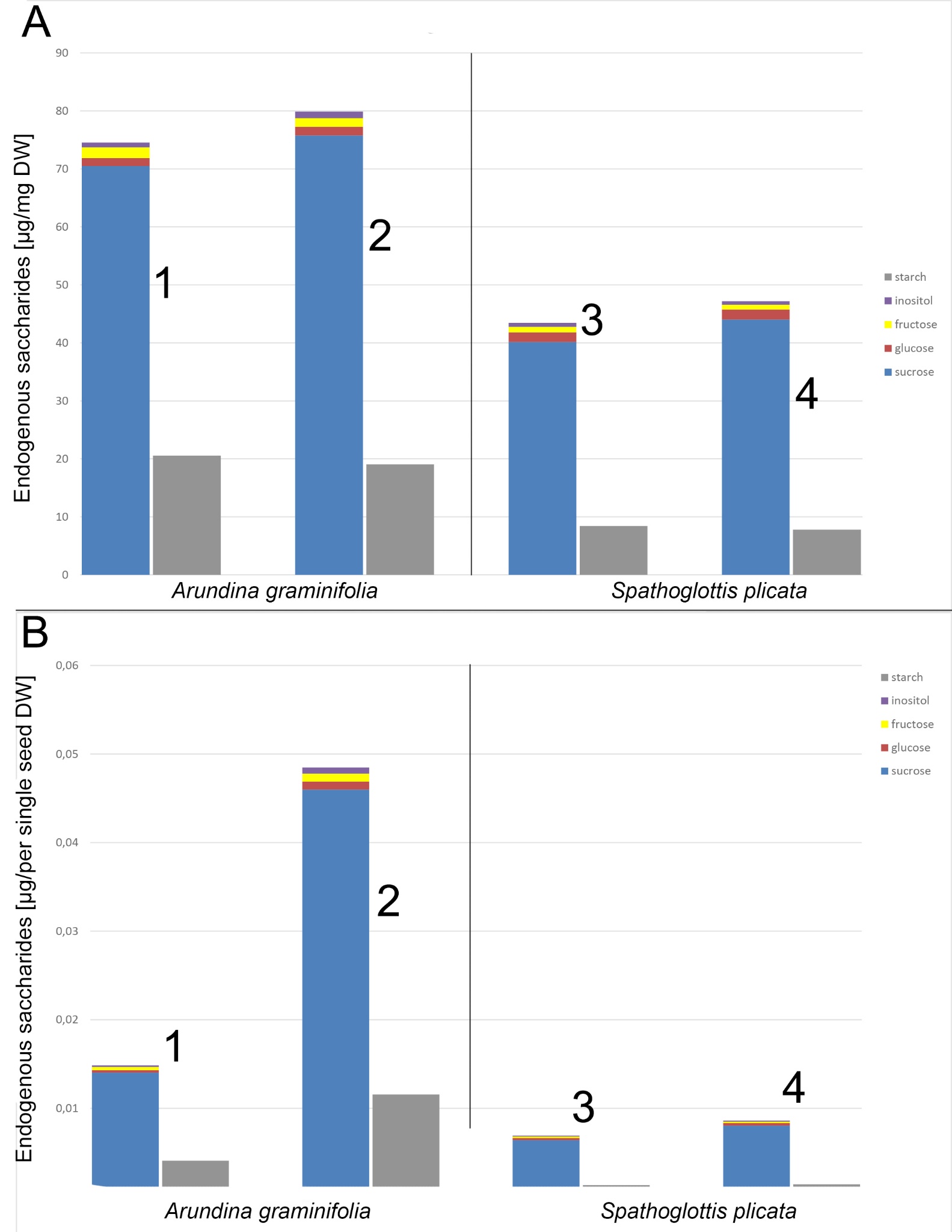


Figure S4: Protocorm formation percentage (protocorms with rhizoids only) of (A) *Arundina graminifolia* and (B) *Spathoglottis plicata* on different carbon sources. Differences between variants were assessed by ANOVA followed by TukeyHSD test. Different letters show differences between variants. P-value for (A) *Arundina graminifolia* was p=0,0005021 and for (B) *Spathoglottis plicata* p=0,002646.
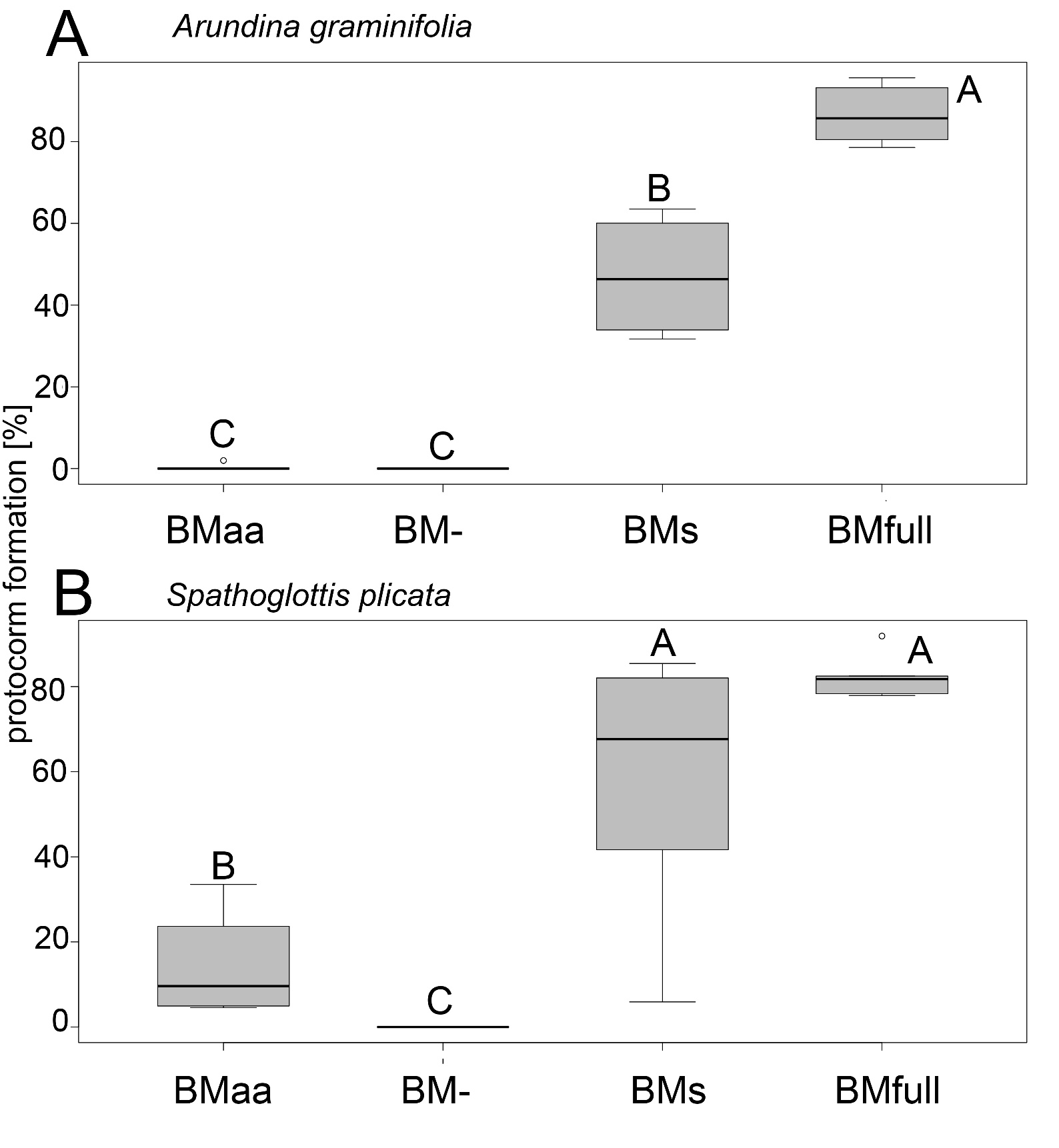


Figure S5: Imbibed dead seeds of (A) *Arundina graminifolia* and (B) *Spathoglottis plicata* on medium with elevated amino acid level “BMaa+”. Scale bar 1 cm.
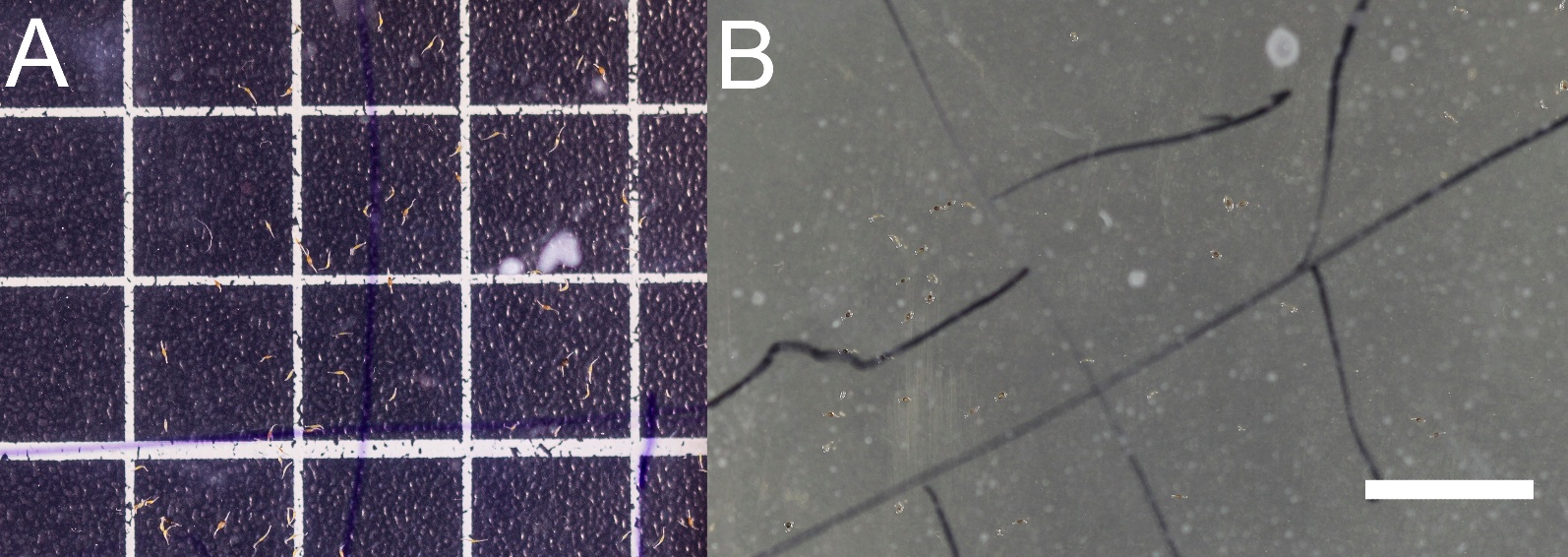


Figure S6: Germination percentage of (A) *Arundina graminifolia* and (B) *Spathoglottis plicata* seeds breaking testa (including protocorms) on different fungal isolates. Different letters show differences between variants according to the pairwise Wilcoxon test. Blue marked are asymbiotic controls, red are Ceratobasidiaceae isolates, yellow Serendipitaceae, pale green European Tulasnellaceae and dark green African (La Réunion) Tulasnellaceae. P-values for (A) *Arundina graminifolia* was p=9,639^e-^*^11^* and (B) *Spathoglottis plicata* p=1,139^e-07^
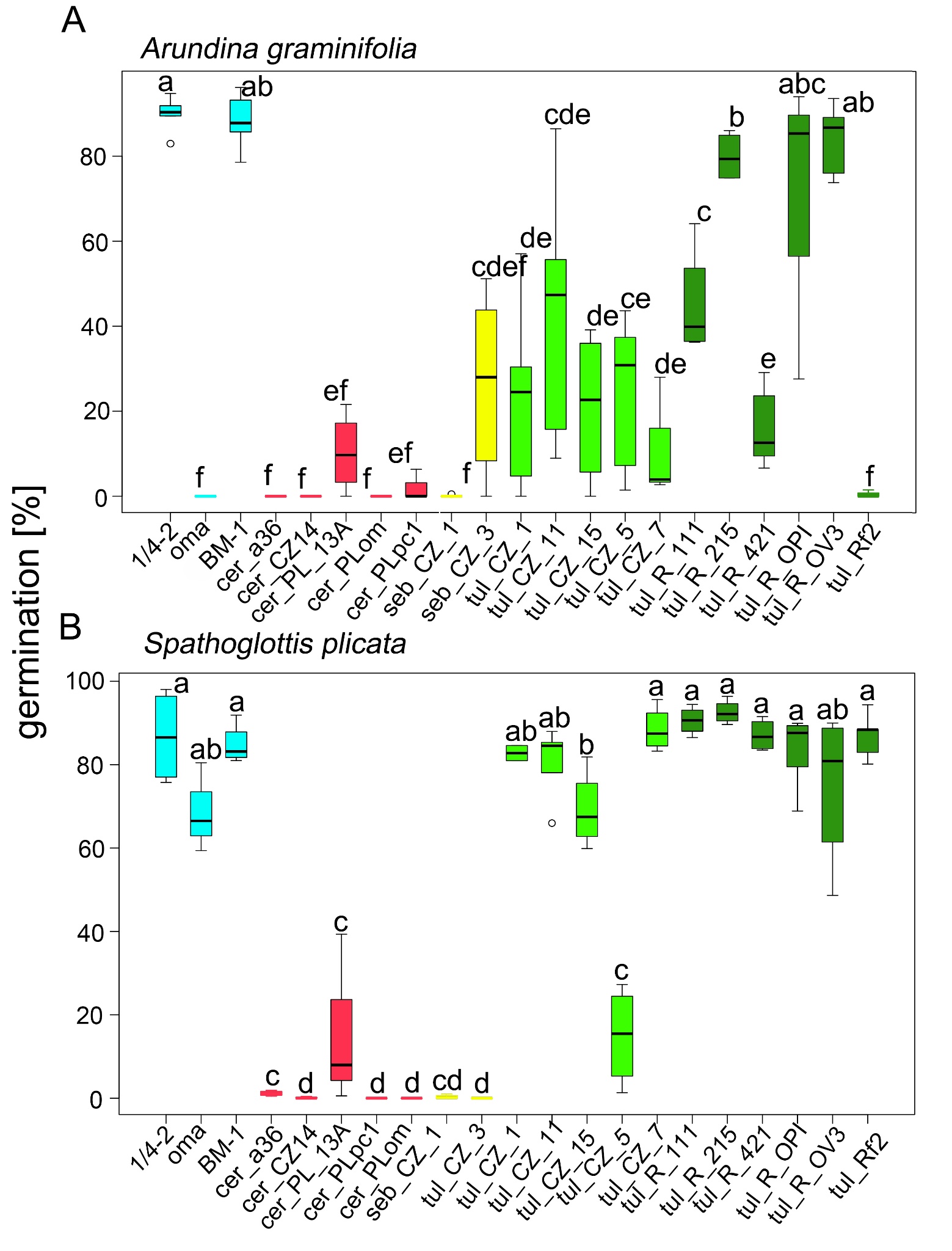

Supplement: Supplementary file 1 — Figure S1: Seeds and protocorms of Arundina graminifolia in all asymbiotic (A, B, U, V, W, X) and symbiotic (C–T) experimental variants tested (A) ¼‐2, (B) oma, (C) cer_a36, (D) cer_PL13, (E) cer_CZ14, (F) cer_PLom, (G) cer_pc1, (H) seb_CZ1, (I) seb_CZ3, (J) tul_CZ1, (K) tul_CZ11, (L) tul_CZ5, (M) tul_CZ15, (N) tul_CZ7, (O) tul_R_111, (P) tul_R_215, (Q) tul_R_411, (R) tul_R_OPI, (S) tul_R_OV3, (T) tul_Rf2, (U) BMs, (V) BM−, (W) BMaa, (X) full. Each photo A–X is 1 × 1 cm big. Figure S2: Seeds and protocorms of Spathoglottis plicata (A) ¼‐2, (B) oma, (C) cer_a36, (D) cer_PL13, (E) cer_CZ14, (F) cer_PLom, (G) seb_CZ1, (H) seb_CZ3, (I) tul_CZ1, (J) tul_CZ11, (K) tul_CZl5, (L) tul_CZ15, (M) tul_CZ7, (N) tul_R_111, (O) tul_R_215, (P) tul_R_411, (Q) tul_R_OPI, (R) tul_R_OV3, (S) tul_Rf2, (T) BMs, (U) BM−, (V) BMaa, (W) BMfull. Each photo A–W is 1 × 1 cm big. Figure S3: Differences in endogenous soluble saccharides and starch contents in μg/mg dry weight (A) and in μg per one seed (B) in A. graminifolia (samples 1, 2) and S. plicata (samples 3, 4). Figure S4: Protocorm formation percentage (protocorms with rhizoids only) of (A) Arundina graminifolia and (B) Spathoglottis plicata on different carbon sources. Differences between variants were assessed by ANOVA followed by TukeyHSD test. Different letters show differences between variants. p‐value for (A) Arundina graminifolia was p = 0.0005021 and for (B) Spathoglottis plicata p = 0.002646. Figure S5: Imbibed dead seeds of (A) Arundina graminifolia and (B) Spathoglottis plicata on medium with elevated amino acid level “BMaa+”. Scale bar 1 cm. Figure S6: Percentage of (A) Arundina graminifolia and (B) Spathoglottis plicata seeds breaking testa (including protocorms) on different fungal isolates. Different letters show differences between variants according to the pairwise Wilcoxon test. Blue marked are asymbiotic controls, red are Ceratobasidiaceae isolates, yellow Serendipitaceae, pale green European Tulasnellaceae and dark g [file ECE3-16-e73805-s001.docx]
